# Evaluation of Xpert MTB/RIF Ultra for the Diagnosis of Extrapulmonary Tuberculosis: A Retrospective Analysis in Saudi Arabia

**DOI:** 10.1007/s44197-023-00150-z

**Published:** 2023-09-14

**Authors:** Mousa J. Slail, Rayan Y. Booq, Ibrahim H. Al-Ahmad, Arwa A. Alharbi, Shafi F. Alharbi, Mutlaq Z. Alotaibi, Abdulaziz M. Aljubran, Ahmad M. Aldossary, Ziad A. Memish, Essam J. Alyamani, Essam A. Tawfik, Abdulwahab Z. Binjomah

**Affiliations:** 1 Tuberculosis Department, Dammam Regional Laboratory, Ministry of Health, Dammam, Saudi Arabia; 2 Wellness and Preventive Medicine Institute, Health Sector, King Abdulaziz City for Science and Technology (KACST), 11442 Riyadh, Saudi Arabia; 3 Mycobacteriology Unit, Riyadh Regional Laboratory, Ministry of Health, 12746 Riyadh, Saudi Arabia; 4 College of Medicine, Alfaisal University, 11533 Riyadh, Saudi Arabia; 5 Research & Innovation Center, King Saud Medical City, Riyadh, Saudi Arabia; 6 Hubert Department School of Public Health, Emory University, Atlanta, USA; 7 Division of Infectious Diseases, Kyung Hee University, Seoul, Korea; 8 Advanced Diagnostics and Therapeutics Institute, Health Sector, King Abdulaziz City for Science and Technology (KACST), 11442 Riyadh, Saudi Arabia

**Keywords:** Extrapulmonary tuberculosis, Pulmonary tuberculosis, Xpert Ultra, Rapid detection, Real-time polymerase chain reaction (RT-PCR), Saudi Arabia

## Abstract

The incidence of extrapulmonary tuberculosis (EPTB) in low- and middle-income countries, as well as, high-income countries has increased over the last two decades. The acid-fast bacillus (AFB) smear test is easy to perform and cost-effective with a quick turnaround time but the test has low sensitivity. Culture remains the gold standard for detecting TB; however, it has low sensitivity and slow bacterial growth patterns, as it may take up to 6 to 8 weeks to grow. Therefore, a rapid detection tool is crucial for the early initiation of treatment and ensuring an improved therapeutic outcome. Here, the Xpert Ultra system was developed as a nucleic acid amplification technique to accelerate the detection of MTB in paucibacillary clinical samples and endorsed by the World Health Organization. From March 2020 to August 2021, Xpert Ultra was evaluated for its sensitivity and specificity against EPTB and compared with those of the routinely used Xpert, culture, and AFB tests in 845 clinical samples in Saudi Arabia. The results indicate the overall sensitivity and specificity of Xpert Ultra to be 91% and 95%, respectively, compared with the Xpert (82% and 99%, respectively) and AFB smear (18% and 100%, respectively) tests. The results also indicated that despite the low microbial loads that were categorized as trace, very low, or low on Xpert Ultra, yet, complete detection was achieved with some sample types (i.e., 100% detection). Consequently, Xpert Ultra has great potential to replace conventional diagnostic approaches as a standard detection method for EPTB.

## Introduction

Tuberculosis (TB) is a leading global health problem that results in significant morbidity and mortality. The infection is caused by the airborne pathogen *Mycobacterium tuberculosis* (MTB) [1–3] and is arguably the leading cause of infectious disease-related mortality worldwide [4]. Since nearly one-third of all active TB cases are underreported and undiagnosed, the rapid detection of MTB is a crucial, life-saving intervention [4]. TB is classified either as pulmonary TB if involving the lung parenchyma, and extrapulmonary TB (EPTB), if involving sites other than the lungs. Several studies have suggested that the site of EPTB varies depending on the geographic location and population. According to the World Health Organization (WHO), EPTB accounts for 16% of all TB cases worldwide [3, 5]. Young children and immunocompromised individuals have a higher risk of EPTB infections [2, 6–8]. Since the advent of HIV infection, EPTB has become more predominant and has been found concurrent with HIV infection in more than 50% of patients [2]. However, the diagnosis of EPTB tends to be more challenging than that of pulmonary TB due to the paucibacillary nature of the disease [1, 2].

TB is generally diagnosed by a microbiological, radiological, or histological approach. The microbiological approach includes smear examination, culture, and molecular-based tests. While culture remains the gold standard laboratory method for diagnosing MTB, it has low sensitivity against EPTB with a low bacillary load and requires 6 or 8 weeks for optimal bacterial growth in a liquid or solid medium, respectively [2, 3]. Culture is also required for drug susceptibility testing (DST). Therefore, the delay in diagnosis may increase the rate of TB disease transmission if drug resistance is present.

The acid-fast bacilli (AFB) smear test can be used as a rapid test (24-h turnaround time) for TB detection [9]. However, the limit of detection (LOD) of the conventional AFB smear technique of fewer than 10,000 bacterial units per milliliter (mL) is difficult to estimate under microscopy [5]. The sensitivity of the AFB smear is reported to be ~ 50% but could be as low as 10–20% for paucibacillary disease [10, 11]. Traditional staining methods, such as Zeihl–Neelsen (ZN), Kinyoun, and Auramine-O, are more cost-effective and might provide rapid results but are similarly limited by a lower sensitivity and specificity than those of the culture and nucleic acid-based methods [12]. Furthermore, several reports have demonstrated that MTB may not be detectable using conventional staining methods [13–16]. Thus, MTB detection using nucleic acid amplification techniques can be considered as more favorable and rapid detection, which are crucial features for early treatment initiation and improved therapeutic outcomes.

In December 2010, the WHO endorsed the implementation of a rapid nucleic acid amplification test for the diagnosis of TB called Xpert MTB/RIF (Cepheid, Sunnyvale, CA, USA) [1, 17, 18]. Xpert is a real-time polymerase chain reaction (RT-PCR) assay that can concurrently detect rifampicin resistance (RR) as an indicator of multi-drug resistant TB (MDR-TB). The assay was designed to use low biosafety level settings for the simultaneous detection of the genetic material of MTB and its mutations, with a turnaround time of < 2 h [1, 17, 19]. The pooled sensitivity of Xpert for culture-positive TB is 98% and 67% for smear-positive and -negative specimens, respectively. In the case of RR-TB, the sensitivity and specificity of Xpert is 95–96% and 98%, respectively [20, 21]. Xpert is also used to diagnose HIV, with a pooled sensitivity of 79–81% regardless of the sputum smear status. A previous report demonstrated that the routine addition of a molecular-based test to the diagnostic algorithm of EPTB has an overall moderate clinical utility, with excellent performance for cold abscess, tuberculous meningitis, and renal TB [22].

The next generation of the Xpert, known as Xpert Ultra, was launched in 2017. It improves upon the performance of the Xpert in smear-negative specimens for better detection of MTB and a higher LOD of 15.6 CFU/mL [23, 24]. The sensitivity of the Xpert Ultra was increased by combining two new PCR assays targeting the multi-copy genes *IS6110* and *IS1081*, a larger DNA reaction chamber, and a transformation from the hemi-nested to the fully-nested PCR reactions. Conversely, the specificity of the Xpert Ultra decreased as a result of enhancing its sensitivity [1, 24]. The WHO endorsement of the Xpert Ultra assay was based on a prospective study that included 129 HIV-positive adults in Uganda with suspected tuberculous meningitis, which was confirmed in 22. The study established the sensitivity of Xpert Ultra at around 95% vs. 45% for Xpert [25]. Nevertheless, a subsequent randomized trial conducted to confirm concurrent HIV infection in 205 patients with tuberculous meningitis showed that Xpert Ultra was not superior to Xpert [26]. This has delayed the official use of Xpert Ultra as the gold standard diagnostic tool rather than the culture for TB detection [26]. Furthermore, studies evaluating the performance of the Xpert Ultra with various EPTB specimens, especially in HIV-negative populations, are limited to certain geographical regions. Therefore, this study evaluated retrospective data from EPTB clinical specimens analyzed at reference TB laboratories in the central (Riyadh City), and eastern (Dammam City) provinces of Saudi Arabia over 18 months (March 2020 to August 2021). This study evaluated the performance and diagnostic accuracy of Xpert Ultra for detecting EPTB and compared it with those of the routinely used culture, AFB, and Xpert tests in different clinical settings. All EPTB specimens were diagnosed by Kinyoun AFB staining, microbiological growth indicator tube (MGIT) culture, MGIT DST, Xpert, and Xpert Ultra, with the MGIT culture test used as the reference standard method.

## Materials and Methods

This study compared the performance of three diagnostic methods to diagnose EPTB in Saudi Arabia based on sample type, age group, gender, and nationality. The experiment in this study was designed in two phases. The first phase included 140 EPTB samples, from different nationalities, to compare Xpert Ultra, Xpert, and AFB staining with the MGIT, whereas the second phase included 845 EPTB samples, in addition to the 140 samples of the first phase, to compare Xpert Ultra and AFB with MGIT.

### Specimens Processing and Laboratory Settings

EPTB clinical samples were obtained from 845 patients suspected of EPTB. Specimen types included cerebrospinal fluid (CSF), tissue biopsies, pus, pleural fluid, gastric aspirate, and urine, among others (Table 1). All samples were collected and managed in modified Biosafety Level 3 laboratories in two regions in Saudi Arabia, i.e., the Mycobacteriology Reference Laboratory in Riyadh City (an ISO 15189 accredited laboratory specializing in MTB detection) and the TB laboratory at the regional laboratory in Dammam City.

**Table 1:** The distribution of EPTB in different clinical specimens

Specimen	Number (*n* = 845)	Percentages (%)
Tissue	377	44.62
Pleural fluid	179	21.18
Pus	83	9.82
Biopsies	41	4.85
CSF	38	4.50
Ascitic fluid	32	3.79
Peritoneal fluid	20	2.37
Pericardial fluid	18	2.13
Gastric aspirate	17	2.01
Synovial fluid	13	1.54
Aspiration fluid	9	1.07
Abscess	8	0.95
FNA	5	0.59
Bone	2	0.24
Stool	1	0.12
Swab	1	0.12
Urine	1	0.12

*CSF* cerebrospinal fluid, *FNA* fine-needle aspirates

Nonsterile clinical specimens were subjected to treatment with MycoPrep decontamination solution (MycoPrep, Becton Dickinson, Sparks, MD, USA) [27]. Briefly, samples were transferred into 50-mL sterile tubes to be decontaminated by adding equal volumes of MycoPrep solution. After 20 min of incubation at room temperature, freshly prepared phosphate buffer saline (PBS; pH 6.8) was added at a final volume of 50 mL to neutralize the samples. Samples were centrifuged at 3000 g for 15 min. The supernatant was decanted, and the concentrated sediment was resuspended into 2–3 mL PBS. The resulting pellets were tested using three methods: (i) Kinyoun smear microscopy; (ii) MGIT using a Bactec 960 instrument (BACTEC MGIT, Becton Dickinson, Sparks, MD, USA) for liquid culture; and (iii) Xpert and Xpert Ultra assays.

### Culturing of EPTB Specimens

All EPTB specimens were inoculated into a liquid culture based on bacterial growth fluorometry. Liquid culture was prepared according to the manufacturer's recommendation. MGIT tubes were supplemented with an enrichment supplement (i.e., OADC) and an antibiotic mixture (i.e., PANTA). The decontaminated/sterile portion (0.5 mL) was inoculated into the MGIT tube. All tubes were incubated in an automated liquid culture BACTEC MGIT 960 system (Becton Dickinson) for a maximum incubation period of 42 days at 35–38 °C.

### Direct Drug Sensitivity Testing of EPTB Specimens

For all positive culture samples, first-line direct DST was performed using Bactec MGIT 960 SIRE and Bactec MGIT 960 pyrazinamide kits (Becton Dickinson) that included isoniazid (INH), rifampicin (RIF), ethambutol, and pyrazinamide anti-tuberculous drugs. Strains resistant to INH and RIF were considered to be MDR.

### Kinyoun Smear Microscopy of EPTB Specimens

AFB smears were prepared according to the manufacturer’s recommendation of the Kinyoun staining kit (Becton Dickinson). Briefly, smears were sprayed with carbol fuchsin for 5 min and then de-stained with sulfuric acid for 2 min. The counterstain was then added to the smears for 1 min. In Kinyoun staining, mycobacteria would appear as red or purple rods against a blue background. The quantification of AFB was reported according to the Centers for Disease Control and Prevention scoring system.

### Xpert MTB/RIF Assay of EPTB Specimens

The Xpert MTB/RIF (Xpert; Cepheid, Sunnyvale, CA, USA) molecular assay was performed as described previously [28]. EPTB clinical specimens were diluted with a sample reagent at a ratio of 1:2 according to the manufacturer's recommendation. These mixtures were vortexed every 5 min during a 15-min incubation period at room temperature. Aliquots (2 mL) of the mixtures were transferred to the Xpert test cartridge. Cartridges were placed into the Xpert instrument that automatically generates results, which were read after 90 min. MTB detection was divided into five categories: high, medium, low, very low, and not detected.

### Xpert MTB/RIF Ultra Assay of EPTB Specimens

The Xpert MTB/RIF Ultra (Xpert Ultra; Cepheid, Sunnyvale, CA, USA) assay was performed as recommended by the manufacturer. EPTB clinical specimens were diluted with a sample reagent at a 1:2 ratio. These mixtures were manually agitated twice for at least 10 s and incubated for 15 min at room temperature. Aliquots (2 mL) of the mixtures were transferred to the Xpert Ultra disposable plastic cartridge. Cartridges were loaded into the Xpert instrument. Xpert Ultra uses a hemi-nested PCR to amplify the RR-determining region (RRDR) of the MTB *rpoB* gene. The results were automatically generated by the instrument within 80 min. MTB detection was divided into six categories (high, medium, low, very low, trace, and not detected). The "trace" category indicates the lowest detectable bacillary burden, which is *IS6110/IS1081* positive but *rpoB* negative. The Xpert Ultra reports RR results as detected or not detected for all categories of MTB-positive samples except for the “trace” category, for which the RR results are reported as “indeterminate,” owing to the very low quantity of MTB DNA.

### Statistical Analysis

All Figures were plotted by Microsoft Excel 2013 software, except for Fig. 5, which was analyzed and plotted by GraphPad Prism 5.00 software (San Diego, CA, USA). Descriptive statistics were used to analyze the study population; normally distributed continuous data were expressed as mean ± standard deviation and non-normally distributed continuous data as median and interquartile range. The analysis of categorical variables was assessed using Pearson’s chi-square test. Comparisons between Xpert and Xpert Ultra assays were performed using the χ^2^ test. *P* < 0.05 was considered statistically significant. The positive predictive value (PPV) and negative predictive value (NPV) were indicated as a percentage chance that the results were true positive and negative, respectively.

## Results

**Fig. 1:**
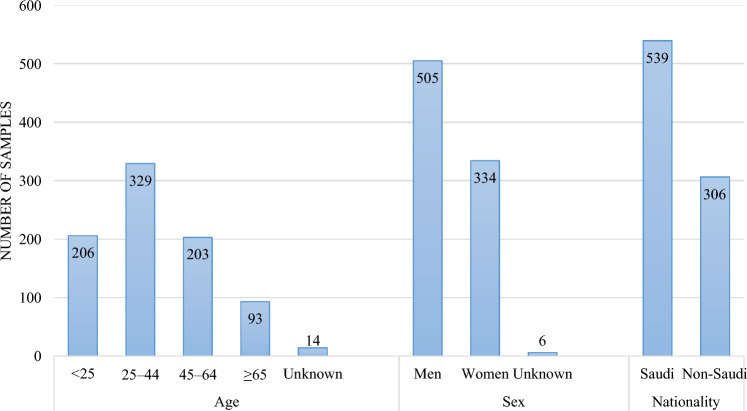
Chart showing patient demographics for *n* = 845 patients

Of the 845 submitted clinical specimens, 682 were negative for bacterial culture (80.71%), while 163 were positive (19.29%). Cases involving Saudis accounted for 64% (*n* = 539), whereas cases with non-Saudis accounted for 36% (*n* = 306), as shown in Fig. 1. More cases were detected in men (60%, *n* = 505) than in women (39%; *n* = 334). The cases were divided into four age groups: < 25 (24%; *n* = 206), 25–44 (39%; *n* = 329), 45–64 (24%; *n* = 203), and ≥ 65 years (11%; *n* = 93).

Although the percentage of samples collected from Saudis was 64% of the total cases in this study (*n* = 845), the number of confirmed cases (positive or infected) was only 39%, compared with the confirmed cases among non-Saudis which was higher (61%).

**Fig. 2:**
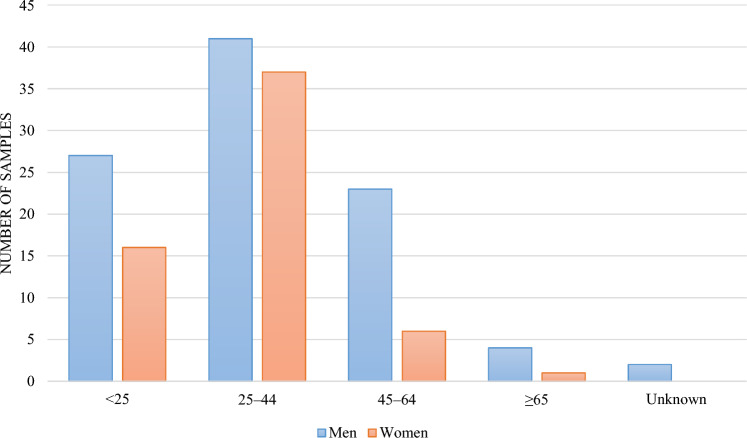
Comparison of TB cases by age group and gender

The average age of the patients was 32 ± 17.1 years. Figure 2 shows the number of positive cases according to age group and gender. The positive cases in the 25–44 age group increased by approximately 50% of the total positive cases, followed by the < 25 age group at 27% and ≥ 45 age group at 22%.

### Diagnostic Performance of Xpert, Xpert Ultra, and AFB Staining Compared with That of MTB Culture

A total of 140 samples from different nationalities were randomly analyzed using Xpert Ultra, Xpert, and AFB staining. Patients with MTB were clinically diagnosed, and the source of the specimens was obtained. Comparatively, the MTB culture found 10 positive samples, whereas Xpert found 10, Xpert Ultra found 16, and AFB staining found 2 samples (Table 2).

**Table 2:** Diagnostic performance of Xpert, Xpert Ultra, and AFB compared with the MGIT culture

	Xpert	Xpert Ultra	AFB	MTB culture
Positive tests	10/140	16/140	2/140	10/140
Sensitivity	82%	91%	18%	–
Specificity	99%	95%	100%	–
PPV	90%	62.5%	100%	–
NPV	98%	99%	93%	–

*PPV* positive predictive value, *NPV* negative predictive value

The differences in diagnostic sensitivity and specificity between Xpert and Xpert Ultra were − 9% and 4%, respectively. The overall sensitivity of the Xpert and Xpert Ultra compared with the reference standard of bacterial culture was 82% and 91%, respectively, whereas the overall specificity of the Xpert and Xpert Ultra was 99% and 95%, respectively (Table 2). The PPV and NPV for the Xpert were 90% and 98%, respectively, compared to 62.5% and 99%, for the Xpert Ultra, whereas, they were 100% and 93%, respectively, for the AFB. Due to the limited positive samples found by AFB staining, more samples were included in the second phase (845 samples) to confirm the low sensitivity (i.e., 18%) of this method in comparison to those of Xpert Ultra and MTB culture.

### Diagnostic Performance of Xpert Ultra and AFB Compared with That of MTB Culture

**Fig. 3:**
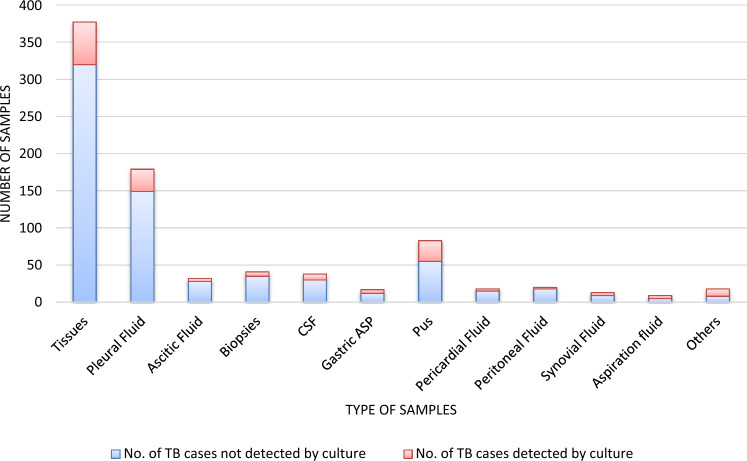
The positive sample distribution of TB compared to the culture test. *CSF* cerebrospinal fluid, *ASP* aspirate

The second phase included 845 samples from 17 different specimens (Table 1) to compare Xpert Ultra and AFB with MTB culture. Figure 3 shows the number of TB-positive samples diagnosed using MTB culture. The number of positive TB isolates confirmed in 163 samples were distributed as follows: 35% tissues; 19% pleural fluid; 17% pus; 5% CSF; 4% biopsies; 3% gastric aspirate; 2% each of ascitic fluid, synovial fluid, aspiration fluid, and pericardial fluid; 1% for peritoneal fluid; and 6% for samples collected from stool, urine, swabs, abscess, fine-needle aspirates (FNA), and bone.

The type of specimen may influence the sensitivity and specificity of TB detection using Xpert Ultra. For instance, diagnosis using gastric aspiration, synovial fluid, and pus specimens showed the highest sensitivity at almost 100%, whereas the detection of the true-positive was the lowest in peritoneal fluid and biopsy samples, which failed detection in nearly half of the samples (50%). Furthermore, the specificity of Xpert Ultra was the highest in CFS samples (97%), followed by peritoneal fluid and biopsy samples (94%). Synovial fluid and gastric aspiration specimens showed the lowest specificity at almost 80%, although they were the highest in terms of sensitivity. The overall sensitivity of Xpert Ultra for all samples was 82%, while the overall specificity was 89% (Table 3).

**Table 3:** Diagnostic performance of Xpert Ultra and AFB staining

Specific specimen	Total number	Xpert Ultra						AFB smear					
		Sensitivity	Specificity	PPV	NPV	NND	CUI +	Sensitivity	Specificity	PPV	NPV	NND	CUI +
Tissue	377	79%	91%	60%	96%	1.44	0.47	18%	100%	91%	87%	5.80	0.16
Pleural fluid	179	83%	86%	54%	96%	1.44	0.45	17%	99%	83%	86%	6.25	0.14
Ascitic fluid	32	75%	93%	60%	96%	1.47	0.45	25%	100%	100%	90%	4.00	0.25
Biopsy	41	50%	94%	60%	92%	2.26	0.30	0%	100%	0%	85%	-	0.00
CSF	38	78%	97%	88%	93%	1.35	0.68	0%	97%	0%	76%	-	0.00
Gastric aspiration	17	100%	82%	75%	100%	1.22	0.75	33%	100%	100%	73%	3.00	0.33
Pus	83	96%	84%	75%	98%	1.25	0.72	50%	100%	100%	80%	2.00	0.50
Pericardial fluid	18	67%	93%	67%	93%	1.67	0.44	0%	100%	0%	83%	-	0.00
Peritoneal fluid	20	50%	94%	50%	94%	2.25	0.25	0%	100%	0%	90%	-	0.00
Synovial fluid	13	100%	78%	67%	100%	1.29	0.67	25%	100%	100%	75%	4.00	0.25
Other aspiration fluids	9	75%	100%	100%	83%	1.33	0.75	0%	100%	0%	56%	-	0.00
Others (stool, urine, swabs, abscess, FNA, bone)	18	80%	50%	67%	67%	3.33	0.53	20%	100%	100%	50%	5.00	0.20
All Specimens (Overall)	845	82%	89%	64%	95%	1.40	0.53	21%	100%	92%	84%	4.75	0.20

*CSF* cerebrospinal fluid, *FNA* fine-needle aspirates, *NND* number needed to diagnose, *CUI+* clinical utility index (< 0.2, poor; > 0.2 to < 0.4, fair; > 0.4 to < 0.6, moderate; > 0.6 to < 0.8, good; and > 0.8 to < 1, excellent); *PPV* positive predictive value, *NPV* negative predictive value

Analysis of the diagnostic performance of AFB staining revealed that its sensitivity and specificity against MGIT culture were 21% and 100%, respectively, and 14% and 100% against Xpert Ultra, respectively. For the smear to show positivity, a high bacterial load (10^3^/mL) is required, as the decrease in the microbial load could reduce the chances of true-positive results [3]. The average microscopic test duration for suspected tuberculosis samples is 30 min, which gives this method superiority over the culture. However, TB detection in low bacterial load samples and the initial stages of the disease cycle is difficult using this method. In addition, the type of sample could significantly influence MTB detection. Luo et al*.* reported the limited diagnostic performance of AFB staining for detecting MTB in CSF samples with a sensitivity of 2.3% [29]. Similarly, in the current study, the diagnostic sensitivity obtained for AFB staining using CSF samples was 0% compared with 78% for Xpert Ultra. Therefore, based on the current findings, using an AFB smear as an initial diagnostic tool for EPTB is not recommended due to its poor sensitivity and clinical utility index.

The NPV for the samples detected by Xpert Ultra were generally higher than the samples tested by the AFP. This was in contrast to the PPV, which was higher for the samples tested by AFP compared to Xpert Ultra, except for the samples obtained from biopsy, CSF, pericardial fluid, peritoneal fluid and other aspiration fluids where the PPV was 0%.

### Diagnostic Performance of Xpert Ultra Based on Microbial Load

**Fig. 4:**
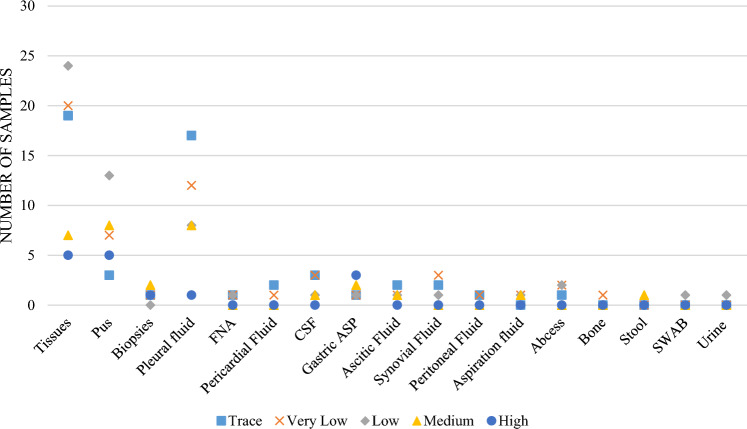
Correlation between the specimen type and sample concentration in *Mycobacterium tuberculosis* detection using Xpert Ultra (trace, very low, low, medium, or high)

MTB detection by Xpert Ultra was further evaluated according to sample concentration or microbial load, in which the microbial genomic replication was detected. This method of detection was compared with the MGIT culture. Positive results on Xpert Ultra were further categorized as trace, very low, low, medium, or high depending on the sample concentration against the bacterial genomic replication detection. The Xpert Ultra produced 636 negative findings compared with 682 for culture and 209 positive findings compared with 163. However, Xpert Ultra obtained 29 false-negative results. Conversely, the Xpert Ultra obtained 132 true-positive results (trace [*n* = 10], very low [*n* = 36], low [*n* = 45], medium [*n* = 28], and high [*n* = 13]) from 163 culture-positive samples and 75 (trace [*n* = 43], very low [*n* = 18], low [*n* = 10], medium [*n* = 2], and high [*n* = 2]) from 682 culture-negative samples. The association between the type and concentration of the samples analyzed by Xpert Ultra was then analyzed (Fig. 4).

**Fig. 5:**
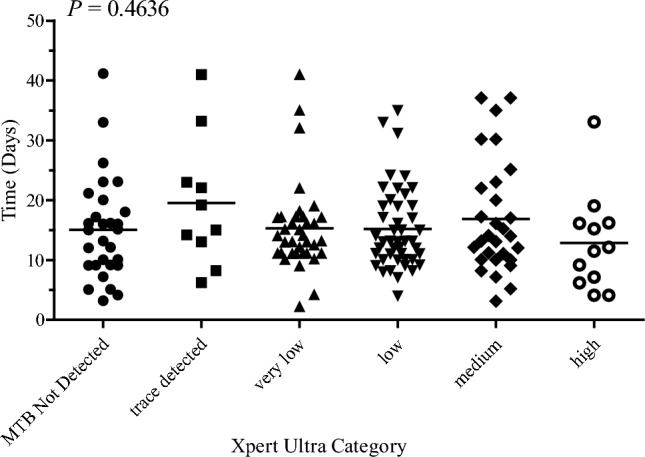
Time to culture positivity compared with the Xpert Ultra results. Outliers were removed, and the dots represent the 25th, 50th, and 75th quartile of data, with upper and lower whiskers representing minimum and maximum values

In evaluating the diagnostic performance of Xpert Ultra, the association between the duration of MGIT culture and the amount of microbial load was analyzed. No significant difference was found between the amount of microbial load and the duration of MGIT culture (*P* = 0.46364). Figure 5 shows a relative decrease in the duration of MGIT culture detection for high microbial loads against the time of culture.

### Diagnostic Ability of Xpert Ultra for RR-MTB

MGIT culture detected 163 MTB-positive samples, 15 (9.20%) of which were identified as drug-resistant using DST. Three of the 15 were classified as RR on Xpert Ultra, whereas the MGIT culture detected 4 samples out of 15. Some samples showed resistance to the other anti-TB drugs, which included streptomycin (in 7 samples), isoniazid (in 5 samples), pyrazinamide (in 3 samples), and ethambutol (in 1 sample) from the overall 15 isolates. These samples were also shown to be resistant to more than one anti-TB drug, which explained why the number of the above resistant samples exceeded the total number of resistant isolates (i.e., 15 samples). Therefore, the prevalence of MDR-TB (resistant to more than one anti-TB drug) was calculated to be 40% of the total number of resistant isolates. On the other hand, the samples detected as antibiotic sensitive were 136 MTB culture-positive samples, while only 15 samples were invalid for antibiotic sensitivity testing. The Xpert Ultra detected only 42 antibiotic-sensitive samples out of the 136 MTB culture samples; all were rifampicin-sensitive. Therefore, the sensitivity and specificity of Xpert Ultra against RR-TB were 100% and 98%, respectively.

## Discussion

The results showed that the sensitivity and specificity of Xpert Ultra in diagnosing patients who are HIV-negative with EPTB were 91% and 95%, respectively, which are superior to those of Xpert (82% and 99%, respectively) and AFB smear (18% and 100%, respectively). Xpert Ultra thus has great potential to replace the conventional diagnostic approaches as the standard detection method for EPTB.

Multiple studies have been conducted to compare Xpert and Xpert Ultra. One prospective multinational study compared the diagnostic accuracy of Xpert with that of Xpert Ultra in sputum. The study findings emphasized the superiority of Xpert Ultra to that of Xpert in diagnosing patients with paucibacillary disease and HIV [30]. The same study showed that the sensitivities of Xpert Ultra and Xpert were 63% and 46%, respectively, for 137 negative smears (90%) and positive culture sputum specimens (77%) in 115 HIV-positive participants. However, the specificities of Xpert Ultra and Xpert were 96% and 98%, respectively, and slightly lower for patients with previous TB (93%). However, both assays performed similarly in detecting RR [30]. Other reports have reported high, moderate, and low sensitivities of Xpert for samples taken from the lymph nodes and pleural fluid, respectively [31, 32]. The pooled sensitivity and specificity of Xpert against RR with EPTB were 95% and 98.7%, respectively [31]. However, Xpert Ultra demonstrated poor sensitivity (44.23%) in a cohort study of 208 patients with pleural TB [33], while it showed a higher sensitivity (90.91%) using osteoarticular TB samples in a study performed in 132 patients with confirmed TB [34].

Wang et al*.* compared the performance of Xpert Ultra to Xpert in the diagnosis of tuberculous meningitis against the culture test for 160 patients in China. They found that Xpert Ultra had a higher sensitivity of 45% than the Xpert (28%) and culture (18%); however, the specificity of all three tests was 100% [35]. Sharma et al*.* also investigated the diagnosis of tuberculous meningitis using Xpert Ultra and compared its performance to that of the Truenat MTB Plus assay using 148 cerebrospinal fluid samples. The findings showed that Xpert Ultra was superior in detecting RR but was comparable to Truenat in terms of tuberculous meningitis diagnosis, with a sensitivity of 67.6% and 78.7%, respectively. Conversely, a higher sensitivity of Xpert Ultra and Truenat were shown at 96% and 85.5%, respectively, in detecting definite tuberculous meningitis [36]. Most recently, Boloko et al*.* evaluated the diagnostic performance of Xpert Ultra using blood samples from 659 patients with suspected HIV-associated TB in South Africa, among whom 447 met the microbiological reference standard for TB diagnosis. Xpert Ultra positively diagnosed 165 (37%) of 447 participants, which consequently contributed to the use of pre-processed blood samples in the study [37]. Another recent study by Costales et al*.* assessed the diagnostic performance of Xpert Ultra on nasopharyngeal samples collected post-mortem from 205 decedents in Tanzania. Xpert Ultra identified 27 (i.e., 13.2%) TB-positive samples at autopsy. It was able to diagnose MTB in 21 (i.e., 77.8%) of the 27 confirmed cases of TB at a sensitivity and specificity of 70.4% and 98.9%, respectively [38].

Few studies have evaluated the diagnostic performance of Xpert Ultra for EPTB using various specimens, especially in HIV-negative populations. Wu et al*.* found that the overall sensitivity and specificity of Xpert Ultra for culture-positive EPTB samples (including CSF, lymph nodes, bone, and urine) were 83.7% and 92%, respectively, and 52.5% and 92%, respectively, for culture-negative EPTB samples [39]. A systematic review and meta-analysis highlighting the performance of Xpert Ultra with TB samples reviewed 16 studies on TB and EPTB and found that seven studies assessing the performance of Xpert Ultra using EPTB samples have shown a pooled sensitivity of 85.1% and specificity of 95.7% [40]. Mekkaoui et al*.* assessed the detection of MTB and RR using Xpert Ultra against other conventional phenotypic techniques for 1120 TB and 461 EPTB clinical samples in Belgium. Overall, Xpert Ultra detected MTB in 223 (14.1%) samples with a sensitivity and specificity of 91.1% and 94.5%, respectively. In the case of smear-negative EPTB samples, the sensitivity of the Xpert Ultra was higher (87.1%) than that of the AFB smear (81.8%). Furthermore, the sensitivity and specificity of the Xpert Ultra for RR were 100% and 99.2%, respectively. Thus, the study demonstrated the reliability of the Xpert Ultra test in diagnosing TB and EPTB in a significantly shorter time than the culture test [3].

Although the percentage of samples collected from Saudis in our study was 64% of the total cases (*n* = 845), the number of confirmed cases (also called positive or infected) was 39% only, compared with the confirmed cases of non-Saudis (61%). These findings are consistent with those of a previous study that reported a lower prevalence of TB among Saudis [41]. Another report conducted over two decades in Saudi Arabia demonstrated that the number of cases recorded among non-Saudis is 2–3 times higher than that of Saudis. In addition, the same study found that, due to the Hajj and Umrah seasons (Islamic religious seasons), increased transmission of the disease from travelers occurs, with a corresponding increase in cases, especially in the Makkah region [42]. These further indicate a higher rate of confirmed EPTB cases in non-Saudis than in the Saudis.

Our study showed that more cases were detected in men (60%; *n* = 505) than women (39%; *n* = 334). This is consistent with a 2019 study that found an increased proportion of men infected with TB than women 1.7 over the past century [43]. Furthermore, the study reported an infection rate of 60% in men compared with 37% in women, with 3% reported as unknown. The high prevalence rate of TB in men compared with that in women may need further investigation at the genetic and physiological levels to determine the association between both genetic and physiological changes to the disease occurrence.

The current study found a higher incidence of TB in the 25–64 age group compared to ≤ 24 and ≥ 65 age groups. A previous study reported that the infection rates of TB increased for the > 45 age group in Saudi Arabia [42], while a more recent study showed that the infection rate was 53% among individuals aged 21–60 years old [44]. The high incidence rate could be due to the reactivation of latent TB particularly in the infection rate among non-Saudi patients exceeding 60% of the total study samples. The number and nationalities of expatriate employees should also be considered in future studies since 10% of the positive cases in this study involved Ethiopian nationals, while 4% were from the Philippines and India.

Our study showed that Xpert Ultra demonstrated higher sensitivity but slightly lower specificity than Xpert for diagnosing TB, which is consistent with the results of several previous studies [45]. Yu et al*.* found that Xpert Ultra showed higher sensitivity (81%) and lower specificity (90%) in FNA samples compared with Xpert (71% and 100%, respectively) [46]. Another study by Signorino et al*.* suggested that the reason for the low specificity of the Xpert Ultra may be attributed to the unsatisfactory quality of the culture method, as a reference test, and needs further investigation [47]. Donovan et al*.* found that Xpert Ultra demonstrated a better performance than Xpert in low TB incidence settings with better rates for false positives and negatives at 90% and 62.5%, respectively. Furthermore, Xpert Ultra had higher sensitivity than the Xpert for tuberculous meningitis [48]. Finally, a cohort study of 23 patients with HIV and definite or probable tuberculous meningitis showed that Xpert Ultra and Xpert had a sensitivity of 69.6% and 43.5%, respectively, and a specificity of 81.8% and 83.3%, respectively [26]. Consequently, the results of Xpert Ultra were adopted in this present study throughout the next phase that included the Xpert Ultra only rather than the Xpert.

Our results showed no significance in the amount of microbial load and duration of MGIT culture, (P = 0.46364). This is in contrast to previous studies reporting an inverse relationship between the microbial load and the duration of MTB growth, i.e., a high microbial load with a lower culture period [49–51]. The reason for the inconsistency between the results could be the splitting of one sample into aliquots for the diagnostic assessments, which may have affected the microbial loads in each aliquot. In contrast, other studies only compared Xpert Ultra with one other alternative method of MTB detection [49–51]. Further studies are required to determine the role and reliability of Xpert Ultra in the diagnosis of TB as a single test.

This present study also demonstrated that despite the low microbial loads that were categorized as trace, very low, or low on Xpert Ultra, complete detection was achieved with some sample types (i.e., 100% detection). For instance, although the microbial loads of MTB from FNA were trace, very low, and low in 3 out of 5 samples, the detection rate and sensitivity were 100%. In contrast, the microbial loads were trace, very low, and low in 37 out of 47 pleural fluid samples, but 5 positive results were not detected. Some negative MTB cultures (i.e., false-negative) from FNA, CSF, synovial fluid, swab, urine, gastric aspirate, and stool samples tested positive on Xpert Ultra. Conversely, some samples had high and medium microbial loads but low detection levels, such as biopsies, which had a PPV of 60%, sensitivity of 50%, and specificity of 94% (Table 3). These results were consistent with those of a very recent study that reported a sensitivity of 45% for pleural biopsy samples [50]. Although pleural fluid is the most frequently used sample type for the diagnosis of pleural tuberculosis, this disease has a very low bacterial load that is below the detection limit of Xpert Ultra and smear microscopy [50].

We found that the sensitivity and specificity of Xpert Ultra against RR-TB were 100% and 98%, respectively. In a recent study conducted in the Makkah region of Saudi Arabia, the prevalence of drug resistance was 17.1% among patients with TB [52], while the prevalence of MDR-TB was 5%. The reason for the increase in the spread of drug resistance in the Makkah region could also be religious seasons, as the majority of pilgrims come from high TB-burdened countries. In another study on Saudi Arabia’s public health perspective of TB, the percentage of MDR strains was 25.49% [41]. In a study on osteoarticular tuberculosis, Xpert Ultra accurately identified all 9 RR and 38 RIF-sensitive cases identified by DST phenotype. Therefore, Xpert Ultra was 100% compatible with the DST phenotype for the detection of RIF resistance [34].

In a large pool study (57 studies) involving several countries with a high PTB burden, Xpert Ultra showed 96% sensitivity and 98% specificity for RR-TB detection [21]. In another study involving patients with EPTB, Xpert Ultra showed almost 100% accuracy (i.e., sensitivity and specificity) for RR-TB detection [31]. The differences in sensitivities reported among the previous studies might be due to genetic variation in the study populations. Moreover, the geographical locations of the sample collection, variances in the sampling technique, and *rpoB* mutations may have also contributed to the variations in diagnostic performance. The assay conditions and technical expertise may also influence the results. Further investigation and evaluation on the cause of the relatively low accuracy of RR-TB detection using both assays is needed to derive conclusive answers.

Our study has some limitations. Despite the large sample size of our study, it included a low number of sample types (e.g., urine and stool). Thus, further investigations should validate the superiority of Xpert Ultra over the alternative methods for EPTB detection using different specimen types. In addition, further studies are needed to evaluate the nature of EPTB samples (i.e., fresh or stored) against the diagnostic accuracy of Xpert Ultra.

In conclusion, the present study indicated that the sensitivity of Xpert Ultra (91%) for EPTB detection was greater than that of Xpert (82%) by 9%, while the specificity was 99% for the Xpert and 95% for the Xpert Ultra. Thus, Xpert Ultra has great potential to replace conventional diagnostic tools as the standard detection method for EPTB and TB. To our knowledge, this study was the largest in terms of sample size (*n* = 845) that compared four diagnostic assays in the Middle East.

## Data Availability

The authors confirm that the data supporting the findings of this study are available within the article.

## Abbreviations

AFB: Acid-fast bacillus
ASP: Aspirate
CSF: Cerebrospinal fluid
CUI: Clinical utility index
EPTB: Extrapulmonary tuberculosis
FNA: Fine-needle aspirates
INH: Isoniazid
LOD: Limit of detection
MGIT: Microbiological growth indicator tube
mL: Milliliter
MDR: Multi-drug resistant
MTB: *Mycobacterium tuberculosis*
NPV: Negative predictive value
NND: Number needed to diagnose
PPV: Positive predictive value
RT-PCR: Real-time polymerase chain reaction
RIF: Rifampicin
RR: Rifampicin resistance
RRDR: RR-determining region
TB: Tuberculosis
WHO: World Health Organization
ZN: Zeihl–Neelsen

## References

[1] Hoel IM, Syre H, Skarstein I, Mustafa T (2020). Xpert MTB/RIF ultra for rapid diagnosis of extrapulmonary tuberculosis in a high-income low-tuberculosis prevalence setting. Sci Rep.

[2] Park M, Kon OM (2021). Use of Xpert MTB/RIF and Xpert Ultra in extrapulmonary tuberculosis. Exp Rev Anti-infect Therapy..

[3] Mekkaoui L, Hallin M, Mouchet F, Payen MC, Maillart E, Clevenbergh P (2021). Performance of Xpert MTB/RIF Ultra for diagnosis of pulmonary and extra-pulmonary tuberculosis, one year of use in a multi-centric hospital laboratory in Brussels, Belgium. PLoS ONE.

[4] Shapiro AE, Ross JM, Yao M, Schiller I, Kohli M, Dendukuri N (2021). Xpert MTB/RIF and Xpert Ultra assays for screening for pulmonary tuberculosis and rifampicin resistance in adults, irrespective of signs or symptoms. Cochrane Database Syst Rev.

[5] World Health Organization (2020) Global tuberculosis report 2020. Geneva: World Health Organization, 2020 Contract No.: ISBN 978-92-4-001313-1

[6] Solovic I, Jonsson J, Korzeniewska-Koseła M, Chiotan D, Pace-Asciak A, Slump E (2013). Challenges in diagnosing extrapulmonary tuberculosis in the European Union, 2011. Eurosurveillance.

[7] Peto HM, Pratt RH, Harrington TA, LoBue PA, Armstrong LR (2009). Epidemiology of extrapulmonary tuberculosis in the United States, 1993–2006. Clin Infect Dis.

[8] Yang Z, Kong Y, Wilson F, Foxman B, Fowler AH, Marrs CF (2004). Identification of risk factors for extrapulmonary tuberculosis. Clin Infect Dis.

[9] Tukvadze N, Kempker RR, Kalandadze I, Kurbatova E, Leonard MK, Apsindzelashvili R (2012). Use of a molecular diagnostic test in AFB smear positive tuberculosis suspects greatly reduces time to detection of multidrug resistant tuberculosis. PLoS ONE.

[10] von Groote-Bidlingmaier F, Koegelenberg CF, Bolliger CT, Chung PK, Rautenbach C, Wasserman E (2013). The yield of different pleural fluid volumes for *Mycobacterium tuberculosis* culture. Thorax.

[11] Wilkinson RJ, Rohlwink U, Misra UK, Van Crevel R, Mai NTH, Dooley KE (2017). Tuberculous meningitis. Nat Rev Neurol.

[12] Zeka AN, Tasbakan S, Cavusoglu C (2011). Evaluation of the GeneXpert MTB/RIF assay for rapid diagnosis of tuberculosis and detection of rifampin resistance in pulmonary and extrapulmonary specimens. J Clin Microbiol.

[13] Binjomah AZA (2014) Studies on the phenotypes of *Mycobacterium tuberculosis* in Sputum: University of Leicester

[14] Lenaerts AJ, Hoff D, Aly S, Ehlers S, Andries K, Cantarero L (2007). Location of persisting mycobacteria in a Guinea pig model of tuberculosis revealed by r207910. Antimicrob Agents Chemotherapy..

[15] Ryan GJ, Hoff DR, Driver ER, Voskuil MI, Gonzalez-Juarrero M, Basaraba RJ (2010). Multiple *M. tuberculosis* phenotypes in mouse and guinea pig lung tissue revealed by a dual-staining approach. PLoS ONE.

[16] Shapiro HM, Hänscheid T (2008). Fuchsin fluorescence in *Mycobacterium tuberculosis*: The Ziehl-Neelsen stain in a new light. J Microbiol Methods.

[17] Boehme CC, Nabeta P, Hillemann D, Nicol MP, Shenai S, Krapp F (2010). Rapid molecular detection of tuberculosis and rifampin resistance. N Engl J Med.

[18] World Health Organization (2011). Rapid implementation of the Xpert MTB/RIF diagnostic test.

[19] Drobniewski F, Nikolayevskyy V, Maxeiner H, Balabanova Y, Casali N, Kontsevaya I (2013). Rapid diagnostics of tuberculosis and drug resistance in the industrialized world: clinical and public health benefits and barriers to implementation. BMC Med.

[20] Pai M, Steingart K, Boehme C, Sohn H, Dendukuri N, Kloda L et al (2013) Xpert® MTB/RIF assay for pulmonary tuberculosis and rifampicin resistance in adults 10.1002/14651858.CD009593.pub2 PMC4470352 23440842

[21] Horne DJ, Kohli M, Zifodya JS, Schiller I, Dendukuri N, Tollefson D, et al (2019) Xpert MTB/RIF and Xpert MTB/RIF Ultra for pulmonary tuberculosis and rifampicin resistance in adults. Cochrane Database Syst Rev (6) 10.1002/14651858.CD009593.pub4 PMC6555588 31173647

[22] Binjomah AZ, Alnimr AM, Zareah SM, Alharbi SF, Alasmari KS, Aldosari KM (2021). The diagnostic impact of implementing a molecular-based algorithm to standard mycobacterial screening at a reference laboratory with an intermediate prevalence for non-respiratory samples. Saudi J Biol Sci..

[23] Organization WH. Frequently asked questions about the WHO Technical Expert Consultation findings on Xpert® MTB/RIF Ultra; 2017. https://www.hoint/tb/publications/2017/XpertUltra/en/

[24] Chakravorty S, Simmons AM, Rowneki M, Parmar H, Cao Y, Ryan J (2017). The new Xpert MTB/RIF Ultra: improving detection of Mycobacterium tuberculosis and resistance to rifampin in an assay suitable for point-of-care testing. MBio.

[25] Bahr NC, Nuwagira E, Evans EE, Cresswell FV, Bystrom PV, Byamukama A (2018). Diagnostic accuracy of Xpert MTB/RIF Ultra for tuberculous meningitis in HIV-infected adults: a prospective cohort study. Lancet Infect Dis.

[26] Donovan J (2020) Improving Diagnosis and Understanding the Pathophysiology of Tuberculous Meningitis: Open University (United Kingdom)

[27] Siddiqi SH, Rüsch-Gerdes S (2006) Procedure manual for BACTEC™ MGIT 960™ TB system. Find Foundation for Innovative New Diagnostics

[28] Helb D, Jones M, Story E, Boehme C, Wallace E, Ho K (2010). Rapid detection of *Mycobacterium tuberculosis* and rifampin resistance by use of on-demand, near-patient technology. J Clin Microbiol.

[29] Luo M, Wang W, Zeng Q, Luo Y, Yang H, Yang X (2018). Tuberculous meningitis diagnosis and treatment in adults: a series of 189 suspected cases. Exp Ther Med.

[30] Dorman SE, Schumacher SG, Alland D, Nabeta P, Armstrong DT, King B (2018). Xpert MTB/RIF Ultra for detection of *Mycobacterium tuberculosis* and rifampicin resistance: a prospective multicentre diagnostic accuracy study. Lancet Infect Dis.

[31] Kohli M, Schiller I, Dendukuri N, Dheda K, Denkinger CM, Schumacher SG et al (2018) Xpert® MTB/RIF assay for extrapulmonary tuberculosis and rifampicin resistance. Cochrane Database Syst Rev (8) 10.1002/14651858.CD012768.pub2 PMC6513199 30148542

[32] Tortoli E, Russo C, Piersimoni C, Mazzola E, Dal Monte P, Pascarella M (2012). Clinical validation of Xpert MTB/RIF for the diagnosis of extrapulmonary tuberculosis. Eur Respir J.

[33] Wang G, Wang S, Yang X, Sun Q, Jiang G, Huo F (2020). Accuracy of Xpert MTB/RIF Ultra for the diagnosis of pleural TB in a multicenter cohort study. Chest.

[34] Sun Q, Wang S, Dong W, Jiang G, Huo F, Ma Y (2019). Diagnostic value of Xpert MTB/RIF Ultra for osteoarticular tuberculosis. J Infect.

[35] Huang M, Wang G, Sun Q, Jiang G, Li W, Ding Z (2021). Diagnostic accuracy of Xpert MTB/RIF Ultra for tuberculous meningitis in a clinical practice setting of China. Diagn Microbiol Infect Dis.

[36] Sharma K, Sharma M, Modi M, Singla N, Sharma A, Sharma A (2021). Comparative analysis of Truenat™ MTB Plus and Xpert(®) Ultra in diagnosing tuberculous meningitis. Int J Tuberc Lung Dis.

[37] Boloko L, Schutz C, Sibiya N, Balfour A, Ward A, Shey M (2022). Xpert Ultra testing of blood in severe HIV-associated tuberculosis to detect and measure *Mycobacterium tuberculosis* blood stream infection: a diagnostic and disease biomarker cohort study. Lancet Microbe.

[38] Costales C, Crump JA, Mremi AR, Amsi PT, Kalengo NH, Kilonzo KG (2022). Performance of Xpert Ultra nasopharyngeal swab for identification of tuberculosis deaths in northern Tanzania. Clin Microbiol Infect.

[39] Wu X, Tan G, Gao R, Yao L, Bi D, Guo Y (2019). Assessment of the Xpert MTB/RIF Ultra assay on rapid diagnosis of extrapulmonary tuberculosis. Int J Infect Dis.

[40] Zhang M, Xue M, He J-q (2020). Diagnostic accuracy of the new Xpert MTB/RIF Ultra for tuberculosis disease: a preliminary systematic review and meta-analysis. Int J Infect Dis.

[41] Saati AA, Khurram M, Faidah H, Haseeb A, Iriti M (2021). A Saudi Arabian public health perspective of tuberculosis. Int J Environ Res Public Health.

[42] Al-Orainey I, Alhedaithy MA, Alanazi AR, Barry MA, Almajid FM (2013). Tuberculosis incidence trends in Saudi Arabia over 20 years: 1991–2010. Ann Thorac Med.

[43] Hertz D, Schneider B (2019). Sex differences in tuberculosis. Semin Immunopathol.

[44] Al-Shahrani MS, Hakami MI, Younis MA, Fan HA, Jeraiby MA, Alraey Y (2021). Prevalence of primary anti-tuberculosis drug resistance at the tertiary center in Saudi Arabia and associated risk factors. Saudi Med J.

[45] Jiang J, Yang J, Shi Y, Jin Y, Tang S, Zhang N (2020). Head-to-head comparison of the diagnostic accuracy of Xpert MTB/RIF and Xpert MTB/RIF Ultra for tuberculosis: a meta-analysis. Infect Dis (Lond).

[46] Yu X, Zhang T, Kong Y, Wang F, Dong L, Han M (2022). Xpert MTB/RIF Ultra outperformed the Xpert assay in tuberculosis lymphadenitis diagnosis: a prospective head-to-head cohort study. Int J Infect Dis.

[47] Signorino C, Votto M, De Filippo M, Marseglia GL, Galli L, Chiappini E (2022). Diagnostic accuracy of Xpert ultra for childhood tuberculosis: a preliminary systematic review and meta-analysis. Pediatr Allergy Immunol.

[48] Donovan J, Phu NH, Dung VTM, Quang TP, Nghia HDT, Oanh PKN (2020). Xpert MTB/RIF Ultra versus Xpert MTB/RIF for the diagnosis of tuberculous meningitis: a prospective, randomised, diagnostic accuracy study. Lancet Infect Dis.

[49] Hodille E, Maisson A, Charlet L, Bauduin C, Genestet C, Fredenucci I (2019). Evaluation of Xpert MTB/RIF Ultra performance for pulmonary tuberculosis diagnosis on smear-negative respiratory samples in a French centre. Eur J Clin Microbiol Infect Dis.

[50] Mansfield M, McLaughlin AM, Roycroft E, Montgomery L, Keane J, Fitzgibbon MM (2022). Diagnostic performance of Xpert MTB/RIF Ultra compared with predecessor test, Xpert MTB/RIF, in a low TB incidence setting: a retrospective service evaluation. Microbiol Spectr..

[51] Menichini M, Lari N, Lupetti A, Rindi L (2020). Evaluation of Xpert MTB/RIF Ultra assay for rapid diagnosis of pulmonary and extra-pulmonary tuberculosis in an Italian center. Eur J Clin Microbiol Infect Dis.

[52] Sambas M, Rabbani U, Al-Gethamy MMM, Surbaya SH, Alharbi FFI, Ahmad RGA (2020). Prevalence and determinants of multidrug-resistant Tuberculosis in Makkah. Saudi Arabia Infect Drug Resist.

